# Association of Early Pregnancy Values of Glycosylated Hemoglobin and the Development of Gestational Diabetes Mellitus

**DOI:** 10.7759/cureus.46685

**Published:** 2023-10-08

**Authors:** Vandana Singh, Shalini Warman

**Affiliations:** 1 Obstetrics and Gynecology, Tata Main Hospital, Jamshedpur, IND

**Keywords:** ogtt, gdm, pregnancy complications, oral glucose tolerance test, high-risk pregnancy, hba1c, gestational diabetes mellitus

## Abstract

Introduction

There is no consensus regarding screening and diagnostic methods for gestational diabetes mellitus (GDM). The present study aimed to evaluate the association between early pregnancy values of glycosylated hemoglobin and the development of gestational diabetes mellitus among pregnant women in a tertiary care hospital in eastern India.

Methods

The prospective cohort study included 200 pregnant women aged between 18 and 35 years in their first trimester (gestational age eight to 13 weeks) attending the antenatal clinics of the study hospital. A glycated hemoglobin (HbA1c) test and a 75-g oral glucose tolerance test (OGTT) test were done in all study participants in their first trimester. Pregnant women with HbA1c ≥6.5% and OGTT ≥140 mg/dl were excluded from the study. In other women, the second trimester (24-28 weeks) and the third trimester OGTT (32-34 weeks) were done to detect gestational diabetes mellitus. Data collection was initiated after the approval of the Information, Education, and Communication (IEC) and relevant authorities. Receiver operating characteristic (ROC) analysis was done to identify the cut-off value of HbA1c that predicted the development of GDM.

Results

The incidence of GDM was 33% among our study participants. The mean HbA1c was significantly higher among women who had GDM (5.4 ± 0.4%) as compared to those who did not develop GDM (4.9 ± 0.2%) (p<0.001). On ROC analysis of HbA1c values to predict the development of GDM, a cut-off value of HbA1c ≥5.25%, irrespective of risk status, was calculated to have 84.8% sensitivity and 62.7% specificity, and among the high-risk group, HbA1c ≥5.15% had 83.3% sensitivity and 97% specificity in predicting GDM. On stratified analysis, a moderately strong positive correlation was demonstrated between HbA1c values and OGTT in the second trimester in both high-risk and low-risk cohorts (p<0.05).

Conclusion

Based on the findings of the present study, HbA1c can be proposed to be a suitable biomarker for GDM prediction, probably not independently but rather as a component of a multi-marker approach for high- and low-risk pregnant groups.

## Introduction

Gestational diabetes mellitus (GDM), by definition, is the development of glucose intolerance with onset or recognition anytime during pregnancy and excludes overt diabetes [1]. GDM is the most prevalent metabolic abnormality during pregnancy and causes severe perinatal outcomes such as macrosomia and obesity, as well as maternal complications, including an increased risk of developing type 2 diabetes mellitus and cardiovascular disease [2]. However, recent studies indicate that, with the use of suitable screening methods, women who are diagnosed and treated for GDM timely have lower perinatal morbidity [3].

There is no consensus regarding screening and diagnostic methods for GDM [4]. The American Diabetes Association (ADA) and the American College of Obstetricians and Gynecologists (ACOG) recommended routine testing of all pregnant women for GDM by glucose challenge or tolerance tests, between the 24th and 28th weeks of gestation, and earlier for those at-risk [5,6]. However, late screening for GDM has been questioned as more recent research supports the idea that GDM-associated fetal overgrowth can begin as early as the first trimester [7], and diagnosis in the latter half of pregnancy or beyond could delay receiving the optimal benefits from medication, exercise, and diet to prevent the development or progression of neonatal and maternal complications [8].

Screening by the oral glucose tolerance test (OGTT) is limited by the need for eight-hour fasting, obtaining at least two blood samples, vomiting, and its high variability, which ultimately leads to failure to complete the test in about 10% of pregnant women [2]. In this context, glycated hemoglobin (HbA1c) has emerged as a potential predictor of GDM in the early stage of pregnancy, owing to its practicability (as it gives an estimate of average glucose over the prior three months) and convenience in clinical practice [9]. However, there is limited evidence on the predictability of HbA1c measured in the first trimester for early prediction of GDM [1,2,10-12].

In light of the dearth of literature in this domain in an Indian setting, the present study was undertaken to evaluate the association between early pregnancy values of glycosylated hemoglobin and the development of gestational diabetes mellitus among pregnant women attending the Department of Obstetrics and Gynecology in a tertiary care hospital in Eastern India.

## Materials and methods

Study design and participants

The present study was a prospective cohort study conducted in the Department of Obstetrics and Gynecology, Tata Main Hospital, Jamshedpur.

The calculation was based on the formula:

$$n=\ frac{(r+1)} {r} \frac{(z_{\alpha } + z_{\beta } ) ^{2}[p_{o}(1-p_{o}) + p_{1}(1-p_{1})] }{(p_{1} - p_{o})^{2}}$$

Where r = allocation unit; zα = z value for desired level of confidence; z𝛽 = z value for desired power; po = proportion of GDM in the low-risk group; and p1 = proportion of GDM in the high-risk group. Assuming a 5% prevalence of diabetes in the low-risk group and 14% in the high-risk group, and considering a 95% confidence limit with an 80% power of study with equal allocation in both study groups, the sample was approximately 181. After inflating the sample size by 10% to make up for the loss in follow-up, the final sample size was calculated to be 200.

Therefore, the study included 200 pregnant women aged between 18 and 35 years in their first trimester (gestational age of eight to 13 weeks) attending the antenatal clinics of the study hospital. Women with pre-pregnancy diabetes, on hypoglycemic drugs, twin pregnancies, and those with hemoglobin ≤8 g/dl were excluded from the study. Based on demographic parameters such as age, weight, body mass index (BMI), family history of diabetes, and history of previous pregnancy, the participants were divided into a low-risk cohort and a high-risk cohort (Table 1). Hundred antenatal women were recruited in each cohort.

**Table 1:** Risk stratification of study cohorts GDM: gestational diabetes mellitus; IUD: intrauterine device; BMI: body mass index

Low-risk cohort	High-risk cohort (presence of atleast one parameter)
Age <25	BMI ≥30 kg/m^2^
No history of diabetes in first-degree relatives	First-degree relatives with type 2 diabetes mellitus
No history of abnormal glucose metabolism	Polycystic ovarian syndrome
Normal weight before pregnancy	Previous pregnancy with GDM
Delivery of normal weight baby in previous pregnancy	Previous delivery of infant weight ≥4.5 kg
	Previous IUD

Study procedure

Data collection was initiated only after obtaining ethical approval from the institutional ethics committee of the hospital. All patients meeting the eligibility criteria were invited to participate after explaining the purpose and procedure of the study. They ensured the anonymity and confidentiality of the data provided by them. Only those patients who provided written informed consent were included in the study. A routine antenatal checkup and relevant history were taken, and an HbA1c test and a 75 g OGTT test were done in all study participants in their first trimester. Pregnant women with HbA1c ≥6.5% and OGTT ≥140 mg/dl were excluded from the study. In other women, the second trimester (24-28 weeks) and the third trimester OGTT (32-34 weeks) were done to detect gestational diabetes mellitus. The participants were followed up until delivery to record the mode of delivery and perinatal outcomes.

Data analysis

The collected data was organized and tabulated in Microsoft Excel 2016 (Microsoft Office 2016 package), and statistical analysis was done using IBM SPSS Statistics for Windows, Version 23.0 (released 2015; IBM Corp., Armonk, New York, United States). The data was analyzed using appropriate statistical tools and represented by various tables, graphs, diagrams, etc. The mean and standard deviation were calculated for quantitative data. Categorical variables were expressed in proportions. Chi-square tests and independent t-tests were used to compare various parameters and outcomes. A receiver operating characteristic (ROC) curve was drawn to find out the cut-off value of HbA1c in the first trimester, which predicts the development of GDM with optimum sensitivity and specificity. The correlation between HbA1c and OGTT values in all trimesters was assessed.

Ethical approval and confidentiality

Approval from the institutional ethics committee of the concerned institution was obtained before starting the study (vide IEC reference no. TMH/AC/IEC/NOV/054/2020 dated 30/11/2020). The confidentiality of the study participants was maintained in all phases of the study.

## Results

Among the 200 consenting pregnant women who participated in the study, the mean age was 24.6 ± 3.9 years, with the majority of the participants in the age group of 18-22 years (40%). The proportion of primigravida mothers was 53%.

Table 2 shows the comparison of clinical characteristics and pregnancy outcomes among the study participants. The mean age in the high-risk group (26.9 ± 3.9 years) was significantly higher than that in the low-risk group (22.3 ± 2.3 years). The mean HbA1c assessed in the first trimester was also significantly higher among the high-risk cohort (5.3 ± 0.4%) as compared to the value in the low-risk cohort (4.8 ± 0.2%).

**Table 2:** Clinical characteristics and pregnancy outcomes of the study participants: comparison between low-risk and high-risk cohorts Values are given as number (percentage) or mean ± SD * P-value was calculated by Chi-square test (categorical variable) and independent t-test (continuous variable) and p<0.05 was considered as statistically significant OGTT: oral glucose tolerance test; HbA1c: glycosylated hemoglobin

Clinical characteristics	High-risk cohort (n=100)	Low-risk cohort (n=100)	p-value*
Age	26.9 ± 3.9	22.3 ± 2.3	<0.001
Gravida			
Primigravida	39 (39.0)	57 (57.0)	0.011
Multigravida	61 (61.0)	43 (43.0)	
OGTT in the first trimester	100.4 ± 15.3	88.2 ± 11.7	<0.001
OGTT in the second trimester	112.8 ± 22.4	91.5 ± 10.1	<0.001
OGTT in the third trimester	141.0 ± 30.6	92.6 ± 15.7	<0.001
HbA1c	5.3 ± 0.4	4.8 ± 0.2	<0.001
Pre-eclampsia	16 (16.0)	6 (6.0)	0.024
Mode of delivery			
Caesarean section	58 (58.0)	44 (44.0)	0.048
Normal vaginal delivery	42 (42.0)	56 (56.0)	
Birth weight	3.1 ± 0.6	3.0 ± 0.4	0.233
Congenital abnormality	2 (2.0)	0 (0.0)	0.155
Shoulder dystocia	16 (16.0)	3 (3.0)	0.002
Respiratory distress syndrome	46 (46.0)	6 (6.0)	<0.001
Neonatal hypoglycemia	36 (36.0)	0 (0.0)	<0.001

In terms of maternal outcomes, the incidence of pre-eclampsia and delivery by caesarean section was significantly higher among high-risk pregnant women (p<0.05). Also, observations of shoulder dystocia, respiratory distress syndrome, and neonatal hypoglycemia were statistically higher in the high-risk group as compared to the low-risk group (<0.05). However, mean birth weight was comparable in both cohorts (p=0.233) (Table 2).

**Figure 1:**
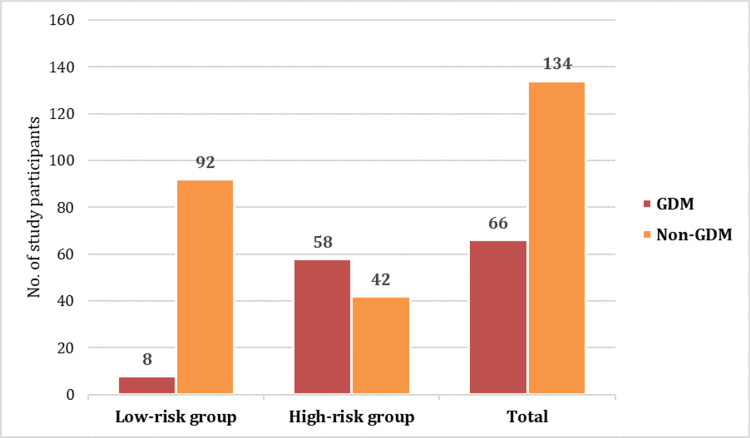
Distribution of study participants according to GDM and non-GDM GDM: gestational diabetes mellitus

The proportion of study participants who developed GDM was 33%, with 8% in the low-risk group and 58% in the high-risk group (Figure 1).

Among the patients who developed GDM, the mean HbA1c in the first trimester was 5.4 ± 0.4%, which was significantly higher than the figure of 4.9 ± 0.2% observed among those who did not develop GDM (p<0.001) (Table 3).

**Table 3:** Comparison of mean of HbA1c in gestational diabetes and non-gestational diabetes participants HbA1c: glycosylated hemoglobin

GDM	Mean ± SD HbA1c	t _|value|_	p-value
Present (n=66)	5.4 ± 0.4	13.53	<0.001
Absent (n=134)	4.9 ± 0.2		

**Figure 2:**
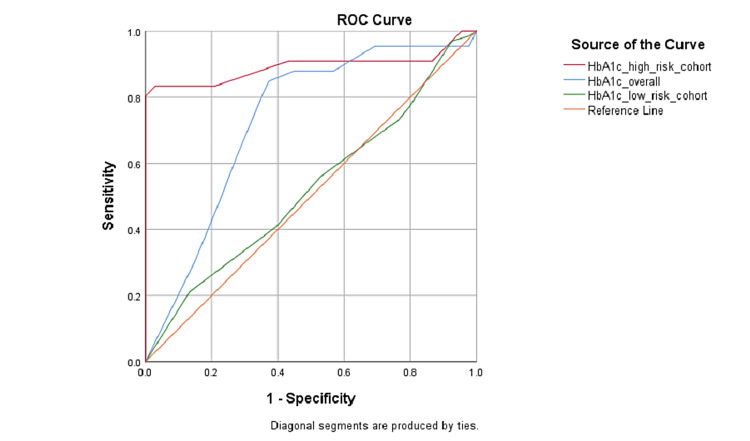
ROC curve for HbA1c in predicting the development of GDM ROC: receiver operating characteristic; HbA1c: glycosylated hemoglobin; GDM: gestational diabetes mellitus

On receiver operating characteristic (ROC) analysis of HbA1c values to predict the development of GDM, a cut-off value of HbA1c ≥5.25%, irrespective of risk status, was calculated to have 84.8% sensitivity and 62.7% specificity (AUC=0.731, p<0.001), and among the high-risk group, HbA1c ≥5.15% had 83.3% sensitivity and 97% specificity (AUC=0.892, p<0.001) in predicting GDM (Figure 2).

On stratified analysis, a moderately strong positive correlation was demonstrated between HbA1c values and OGTT in the second trimester in both high-risk and low-risk cohorts, which was statistically significant. OGTT values in the third trimester showed a moderately strong positive correlation with HbA1c only in the high-risk participants, with p<0.05 (Table 4).

**Table 4:** Correlation between HbA1c and OGTT values in first, second and third trimesters * P-value was calculated by correlation analysis (Spearman’s) and p<0.05 was considered as statistically significant OGTT: oral glucose tolerance test; HbA1c: glycosylated hemoglobin

Group	Spearman’s correlation coefficient	Asymptotic Standard Error	p-value*
First trimester			
High-risk cohort	.132	.107	0.187
Low-risk cohort	.083	.102	0.416
Second trimester			
High-risk cohort	.454	.088	<0.001
Low-risk cohort	.277	.095	0.006
Third trimester			
High-risk cohort	.590	.079	<0.001
Low-risk cohort	-.048	.107	0.636

## Discussion

In the present study, the incidence of GDM was 33%. It must be kept in mind that the present study included an equal number of pregnant women who were at low risk and at high risk of developing GDM based on clinical parameters and history-taking. Bahl S et al., after analyzing data from the Women and Infants Integrated Interventions for Growth Study (WINGS) in North India, reported the incidence of GDM as 19.2% [13]. Another study by Chanda S et al. reported a 16.67% prevalence of GDM in rural areas of north-east India [14]. Despite wide variability across the country and regardless of the criteria used, it is evident that the incidence of GDM is comparatively higher in Indian women as compared to the global picture [14].

There is considerable interest in the ability to predict the risk of GDM as early as possible. Predicting GDM in the first trimester of pregnancy would enable a prompt response to mitigate adverse pregnancy outcomes linked to GDM in the short and long run. The focus of current diagnostic recommendations for the first trimester is on ruling out undiscovered pregestational diabetes or other types of dysglycemia [15]. However, the majority of the women don't seem to have blood sugar levels high enough to meet the requirements for impaired fasting or impaired glucose tolerance [16]. Fasting and postprandial glucose concentrations are typically lower in the early stages of pregnancy compared to those in non-pregnant women [16]. Elevated fasting or postprandial plasma glucose levels at this point in pregnancy may indicate the presence of dysglycemia; however, the standards for identifying abnormally high glucose levels at this point have not yet been developed or demonstrated in prior studies [15]. The present study utilized early values of glycated hemoglobin (HbA1c) in the first trimester and correlated the same with OGTT values recorded in each trimester or by the study participants. Also, an attempt was made to identify the optimum cut-off value of HbA1c in the first trimester, which predicted the development of GDM with the best sensitivity and specificity.

The diagnosis of GDM was solely based on OGTT values conducted in the second and third trimesters for all the study participants. It was observed that although the mean HbA1c and mean OGTT levels in the first trimester were significantly higher in the high-risk group as compared to the low-risk group, there was no significant correlation between the two parameters. A moderately strong correlation between OGTT in the second trimester and HbA1c values in both cohorts points to the supposition that HbA1c levels in the first trimester might provide an early indication of the potential blood glucose trends that pregnant women might develop subsequently with the progression of pregnancy. However, pregnant women who were already diagnosed with GDM in the second trimester were advised on diet and lifestyle modification along with insulin, which would have lowered blood glucose levels and resulted in normal or near-normal OGTT levels in the third trimester. This may have been the reason for the loss of correlation between HbA1c levels and OGTT values in the third trimester among the low-risk cohort. However, significantly higher OGTT values in the third trimester in the high-risk cohort might be because of the development of GDM among this group in the latter stage of pregnancy. Overall, the mean HbA1c was significantly higher among women who develop GDM (5.4 ± 0.4%) as compared to those who do not develop GDM (4.9 ± 0.2%).

Our observations highlighted that HbA1c in the first trimester was capable of predicting the development of GDM, irrespective of the risk stratification of pregnant mothers. The ROC analysis showed a significant relationship between the HbA1c value and the occurrence of GDM. The ROC curve drawn from HbA1c values in the overall population had an area under curve of 0.731 (p<0.001). The cut-off value of HbA1c ≥5.25% was calculated to have 84.8% sensitivity and 62.7% specificity in predicting GDM. However, in the high-risk cohort, a cut-off value of HbA1c ≥5.15% had 83.3% sensitivity and 97% specificity in predicting GDM (AUC=0.892, p<0.001). A study by Valadan M et al. reported an HbA1c cut-off value of 4.85% for ruling out GDM with a sensitivity and specificity of 92.2% and 32.8%, respectively, while the sensitivity and specificity for diagnosing GDM at an HbA1c ≥5.45% were 54.8 and 96.8%, respectively [2]. Hinkle SN et al. observed a sensitivity of 62% and a specificity of 82% for the HbA1c cut-off value of 5.3% [10]. Çetin C et al. identified HbA1c ≥5.33% to have 71.9% sensitivity and 82.8% specificity for GDM diagnosis [11]. Moreover, in a systematic review by Kattini R et al., HbA1c >5.7% has been reported to have high specificity for predicting the development of GDM during pregnancy [17]. While the aforementioned studies informed HbA1c cut-offs for the general pregnant population, our study identified the potential values of HbA1c in the first trimester to predict GDM in both the general antenatal women as well as that among the high-risk pregnant women. To our best knowledge, Amylidi S et al. was the only other study that reported first-trimester HbA1c >6.0% as predictive of GDM among pregnant women at high risk for GDM [18].

The present work is conducted for the first time in an Indian hospital setting with an acceptable sample size. Our study findings add to the growing pool of evidence in favor of HbA1c as a simple pragmatic tool to identify pregnant women in the first trimester who are at risk of developing GDM. However, in a developing country like India, given the cost-benefit of early identification of women at risk of developing GDM, conducting routine HbA1c testing for all pregnant mothers might help curb the burden of GDM and its complications in the long run. Since the present study was conducted in a single tertiary care center with a limited sample size, our results need to be validated in other studies with a larger sample size conducted across the nation. Furthermore, physiological hydremia during pregnancy, anemia, reduced gut motility, increased red cell turnover, and nutritional variations are factors that can considerably affect the HbA1c value [19]. Besides, enhanced hepatic glucogenesis with the progression of pregnancy, along with a concurrent increase in insulin resistance, leads to a physiological surge in insulin secretion [20]. For these reasons, there is no consensus on using HbA1c for the diagnosis of GDM to date.

## Conclusions

Early prediction of gestational diabetes mellitus in pregnant mothers, both at high-risk and low-risk of developing GDM, carries immense potential to curb the adverse effects on the baby and the mother. Based on the findings of the present study, HbA1c can be proposed to be a suitable biomarker for GDM prediction, probably not independently but rather as a component of a multi-marker approach for high-risk pregnant groups. However, our observations were based on a relatively smaller sample size and conducted in a single center, warranting further large-scale multi-centric prospective studies to validate our findings. Nevertheless, early screening of women at risk of developing gestational diabetes mellitus and the institution of appropriate treatment will have a favorable impact on pregnancy outcomes.
